# A patient-reported questionnaire developed in a German early arthritis cohort to assess periodontitis in patients with rheumatoid arthritis

**DOI:** 10.1186/s13075-019-1982-z

**Published:** 2019-08-29

**Authors:** Johanna Callhoff, Thomas Dietrich, Mariya Chubrieva, Jens Klotsche, Angela Zink

**Affiliations:** 1 Epidemiology Unit, German Rheumatism Research Centre, Charitéplatz 1, Berlin, Germany; 2 Department of Oral Surgery, The School of Dentistry, University of Birmingham, Birmingham, UK; 3 Department of Rheumatology and Clinical Immunology, Charité University Hospital, Berlin, Germany

**Keywords:** Periodontitis, Rheumatoid arthritis, Self-reported questionnaire, Validation

## Abstract

**Background:**

The aim of this study was to develop a patient-reported questionnaire that is suitable to detect periodontitis (PD) in patients with rheumatoid arthritis (RA).

**Methods:**

A self-reported questionnaire containing 12 items potentially relevant to PD and dentists’ semiquantitative assessment of PD (no/mild/moderate/severe) was obtained from 353 patients from an early arthritis cohort. Available radiographs (*n* = 253) and blinded assessment of 3 independent dentists were used for validation. By defining the dentists’ assessment as the reference standard, relevant questionnaire items were identified with factor analysis methods. Receiver operator characteristic (ROC) plots were used to determine sensitivities and specificities to detect PD in varying severity. Ordinal regression models were used to determine the coefficients for the final score.

**Results:**

Seventy percent had at least mild PD. The items from the questionnaire correlating best with the dentists’ assessment were selected for a final 6-item score (number of teeth, gum pockets, receding gums, loose teeth, receding jaw bone and tooth extractions and age). For the detection of any/moderate/severe PD, the bias-corrected areas under the curve (AUC) were 0.81/0.83/0.90. Sensitivity to detect mild PD was 85% and specificity 57%. Very high specificity was achieved for the detection of severe PD with 99% at the cost of low sensitivity (28%).

**Conclusions:**

This patient-reported six-item score has moderate diagnostic properties to study PD in RA patients in epidemiological settings. We propose to use the score as a measure of periodontitis without applying cut-off values.

## Introduction

Over the past years, the association between chronic periodontitis (PD) and rheumatoid arthritis (RA) has received considerable attention [1–5]. In a systematic review of studies on the association of PD and RA [6], Kaur et al. reported good evidence for the association between PD and tooth loss and attachment loss in patients with RA. They also discuss several models for the “interplay between PD and RA”, which include the possibilities that periodontitis precedes RA, that there are common underlying inflammatory pathways and that RA and PD exacerbate each other [6].

In a case-control study with 22 RA patients and 22 healthy controls, Wolff et al. confirmed evidence that patients with RA suffer from a higher risk of periodontal attachment loss [7]. Large epidemiological studies could help to gain further knowledge on the association of PD and parameters of disease activity in RA. However, it may not always be feasible to include the assessment of the periodontal status from trained study dentists in large epidemiological settings as was the case in the studies performed by Choi et al. [8] and Ayravainen et al. [3]. Therefore, a self-reported questionnaire would be helpful to assess PD in patients with RA. Several self-reported patient questionnaires have been developed in the past in various non-RA-specific populations with reasonable validity [9–12]. Coburn et al. [13] published a self-reported PD questionnaire that was evaluated in 617 patients with RA and osteoarthritis. This questionnaire included 6 questions on the periodontal status as well as sex, age, education and smoking behaviour and also showed moderate validity.

Taking into account the previous work by Dietrich et al. [11] and others [9, 10, 12], our aim was to develop a simple patient-reported questionnaire for PD that can be used for studying the relationship of RA and PD in epidemiological settings and to validate it in a large cohort of patients with RA.

## Patients and methods

### Early arthritis cohort

Patients from the early arthritis cohort study Course And Prognosis of Early Arthritis (CAPEA) were asked to participate in this project. CAPEA is a prospective, multicentre, non-interventional, observational study in which patients were enrolled between 2010 and 2013 [14]. Eligible patients had arthritis for less than 6 months. They were consecutively enrolled in rheumatology clinics and practices in Germany and observed for 2 years in order to investigate the prognostic value of early symptoms for the development of a chronic course of disease. Ethical approval for CAPEA was obtained from the Ethics Committee of the Charité University Medicine, Berlin, in May 2009 with an amendment for the periodontitis project in May 2012.

### Patient-reported questionnaire on periodontitis

All patients enrolled in CAPEA until January 2013 were sent a questionnaire including 12 questions about their PD status and other items considering dental replacement, comorbidities, current medication and pain. The questionnaire items were as follows: “number of teeth” (0–28), “receding jaw bone” (0, no; 1, yes), “receding gums” (0, no; 1, at up to 3 teeth; 2, at 4 to 10 teeth; 3, at over 10 teeth), “presence of gum pockets” (0, none; 1, at up to 3 teeth; 2, at 4 to 10 teeth; 3, at over 10 teeth), “loose teeth” (0, no, never; 1, I had loose teeth in the past; 2, yes, I currently have loose teeth), “tooth extractions because of inflammation and deep gum pockets” (0, no; 1, at up to 3 teeth; 2, at 4 to 10 teeth; 3, at more than 10 teeth), “more dentist visits because of inflammation than because of caries” (0, no; 1, yes), “more tooth/gum problems than other persons of the same age and sex” (0, less than others or comparable to others; 1, more than others; 2, a lot more than others), “inflammation of the gums/bleeding” (0, never; 1, every few years; 2, in many years; 3, (nearly) every year), “magnitude of suffering from dental problems in total during the last 6 months” (0, not at all; 1, a little bit; 2, quite a bit; 3, severe problems), “cold- or heat sensitivity” (0, no, never; 1, yes, in the past; 2, yes, currently) and “use of antibiotics to treat inflammation in the jaw bone” (0, never; 1, once; 2, two to five times; 3, more than five times). Most of the questions were illustrated with pictures to demonstrate the appearance of a radiograph with receding jawbone for example. The questionnaire is available from the authors upon request.

### Dentists’ assessment

Patients were asked for the permission to contact their dentists. For all patients who returned a written consent, their dentists were then contacted by mail. They were asked to report whether or not the patient had been diagnosed with PD and to assess the PD status semiquantitatively with the possible answers “no”, “mild (< 30% bone loss)”, “moderate (30–50% bone loss)” or “severe PD (> 50% bone loss)”. Additionally, the number of teeth was reported. Furthermore, the dentists were asked to send any radiographs not older than 5 years for evaluation, if available.

The obtained radiographs were scored independently by three dentists at the School of Dentistry at the University of Birmingham, UK. The dentists were blinded to the clinical data of the patients. Disagreements were resolved by discussion. The confidence in the diagnosis of PD based on the available radiographs was rated as “certain”, “pretty certain” or “uncertain”.

The PD status reported by the patients’ dentists was defined as the reference standard for PD for all analyses.

### Statistical analysis

Correlations between the patient-reported items, the dentists’ assessment and the blinded external assessment of the radiographs were analysed using Spearman’s correlation coefficient. Confirmatory factor analysis was used to test the one-dimensional factor structure of the questionnaire. Items with similar content may result in correlated measurement errors [15] as indicated by large modification indices. Therefore, correlated residuals were assumed in the confirmatory factor model to avoid this method error. The evaluation of the model fit was based on the cut-offs as recommended by Hu and Bentler [16] (root mean square error of approximation (RMSEA) ≤ 0.06, comparative fit index/Tucker-Lewis index (CFI/TLI) ≥ 0.9).

These items were used to calculate a final score for the detection of PD. Since age strongly correlates with the number of teeth and the probability to have PD, we always included age in the score [1].

The diagnostic properties of the score were evaluated by determining the sensitivity, specificity and the area under the receiver operator characteristic curve (AUC). Possible values for the AUC range from 0.5 to 1: 0.5 meaning a random classification of patients as having PD or not and 1 meaning perfect discrimination of the score between the groups. As the PD status was not assessed binary but with several levels of severity, different classifications of patients were performed. This resulted in three binary classifications of PD status: no versus mild/moderate/severe PD, no/mild versus moderate/severe PD and no/mild/moderate versus severe PD. To include all classifications of PD into a single model, an ordinal regression was performed so that it was possible to use the resulting score to assign patients to the most likely level of PD without having to choose which severity of PD should be detected.

### Correction for overoptimism

The AUCs resulting from applying the model based on the whole dataset on the same data are likely too optimistic. We corrected for this overoptimism with bootstrap methodology. For 500 bootstrap samples of the size of the original dataset, models for the PD score were estimated. The resulting models were applied to both the original dataset and the respective bootstrap samples. Differences in the resulting AUCs were calculated, resulting in an estimator for the mean overoptimism. This estimator was subtracted from the original AUCs, resulting in bias-corrected AUCs. Additionally, the models based on the dentist’s assessment of PD were applied to the subsample of patients with a radiographic assessment of PD, using this as the reference standard.

## Results

### Study participation and baseline characteristics

**Fig. 1:**
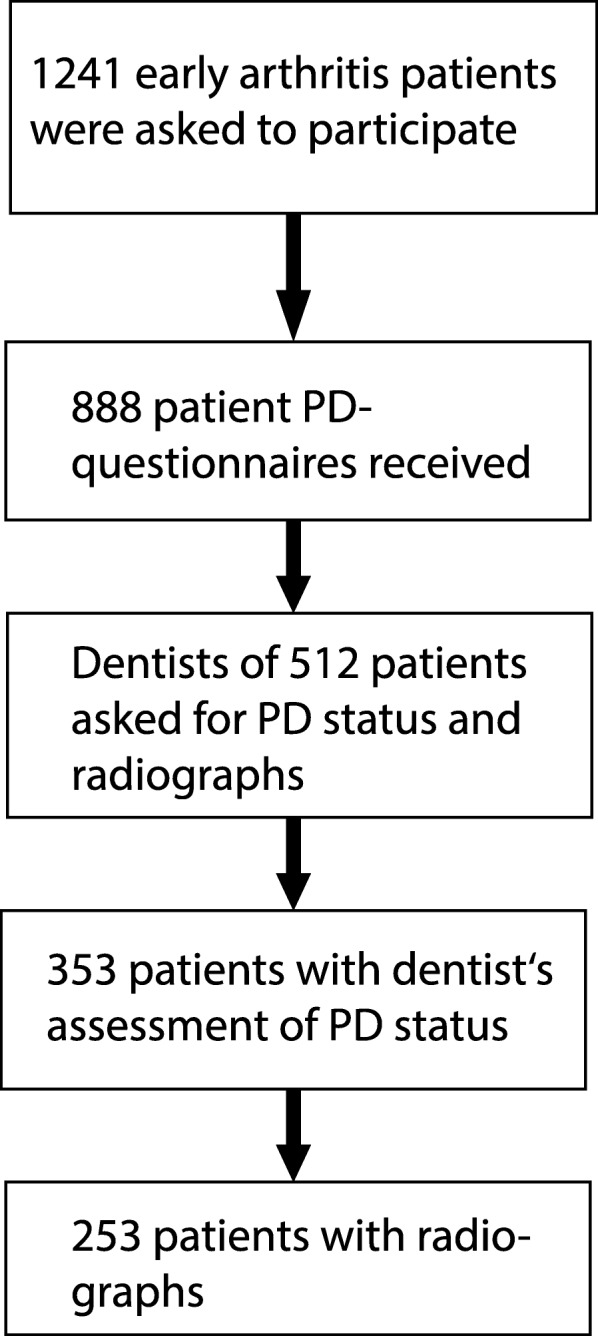
Flowchart of participants who are included in the different analyses

A total of 512 patients completed the patient questionnaire and gave permission to contact their dentists. We received 353 data sets with the dentist’s assessments of the PD status and 253 data sets with additional radiographs. Radiographs of 4 patients were excluded due to insufficient quality. Figure 1 shows a flowchart of the respective patient numbers. The clinical characteristics at baseline of the different patient groups are shown in Table 1. The subgroups were comparable to the CAPEA cohort except for a slightly higher mean number of teeth in the patients with available radiographs.

**Table 1:** Baseline characteristics

Variable	All CAPEA patients (*n* = 1241)	PD module patients (*n* = 512)	Patients with dentist’s assessment (*n* = 353)	Patients with radiograph (*n* = 253)
Age, years	56.2 (14.3)	56.3 (14)	55.8 (13.3)	55.8 (13.3)
Sex, female	65% (821)	67% (575)	64% (250)	67% (170)
DAS28 ESR	4.7 (1.4)	4.8 (1.4)	4.7 (1.4)	4.7 (1.4)
ESR, mm/h	31.2 (23.4)	31.8 (23.7)	31 (22.9)	30 (22)
CRP, mg/l	18.9 (31.9)	17.8 (28.4)	16.7 (22.3)	15.5 (21.4)
Number of teeth	19.2 (9.6)	19.4 (9.6)	20.8 (8.3)	21.1 (7.7)
SJC28	6 (5.2)	6 (5.2)	5.6 (4.8)	5.4 (4.7)
TJC28	9.7 (6.2)	9.5 (6)	9.2 (5.9)	9.1 (5.8)
RF positive	43% (539)	43% (370)	44% (169)	40% (103)
Anti-CCP positive	39% (493)	38% (331)	40% (157)	36% (93)
Currently smoking	33% (413)	29% (254)	27% (105)	25% (65)

Baseline characteristics of all patients from the early arthritis cohort CAPEA, patients who completed the PD-module, patients with additional dentist’s assessment of PD and patients with radiographs

*CAPEA* Course And Prognosis of Early Arthritis, *PD* periodontitis, *RF* rheumatoid factor, *SJC* swollen joint count, *TJC* tender joint count, *ESR* erythrocyte sedimentation rate, *DAS28* Disease Activity Score including 28 joints, *CRP* C-reactive protein

### PD assessments

According to their dentists, 30% of the patients had no, 33% mild, 26% moderate and 11% severe PD. Of the 253 patients with radiographic evaluations, 23% had no, 25% mild, 29% moderate and 23% severe PD. For 41% of the patients, the three independent dentists rated the security of their PD assessment as certain; in 49% of cases, they were moderately certain; and in 11% of cases, they were uncertain, meaning that at least two dentists rated the PD status of the respective patients as uncertain. Certainty was higher for the assessment of no or severe PD than for the assessment of mild or moderate PD.

The correlations between the patient-reported items and the dentists’ assessments of PD are shown in Table 2. They were highest for the patient-reported number of teeth, receding jaw bone, receding gums, presence of gum pockets and loose teeth. Those items also correlated highest with the independent assessment of PD via radiographs. The strength of the correlation is only moderate with the highest correlation coefficient of − 0.49 between the number of teeth and the dentist’s assessment of PD.

**Table 2:** Correlations of questionnaire items with PD assessments

Patient questionnaire item	Number of missing values	Spearman’s corr. with dentist’s assessment	Spearman’s corr. with assessment of radiographs
*Number of teeth*	22	− 0.49	− 0.40
*Receding jaw bone*	25	0.41	0.43
*Receding gums*	14	0.37	0.33
*Presence of gum pockets*	16	0.36	0.31
*Loose teeth*	9	0.36	0.36
*Tooth extractions because of inflammation and deep gum pockets*	13	0.27	0.22
More dentist visits because of inflammation than because of caries	12	0.26	0.17
More tooth/gum problems than other persons of the same age and sex	22	0.19	0.10
Inflammation of the gums/bleeding	8	0.12	0.08
Magnitude of suffering from dental problems	6	0.11	0.07
Cold- or heat sensitivity	6	0.09	0.08
Use of antibiotics	6	0.08	0.13

Correlations of questionnaire items with dentists’ assessment of PD and with an independent assessment of PD via radiographs. Items in italics were found to be the most suitable to detect PD via factor analysis and are included in the final score

### Selection of variables for the patient-reported PD score

The factor structure of two models was tested by confirmatory factor analysis (CFA): (a) a one-factor model in which all items were included and (b) a one-factor model in which six items were included (number of teeth, receding jaw bone, receding gums, presence of gum pockets, loose teeth, tooth extractions because of inflammation and deep gum pockets), which correlated among each other in preliminary analyses. The CFA including all questionnaire items did not result in an acceptable model fit (RMSEA = 0.096, CFI = 0.86, TLI = 0.88, WRMR = 0.96). The model that included six selected items did not fit the data well (RMSEA = 0.148, CFI = 0.89, TLI = 0.82, WRMR = 1.05). The modification indices suggested correlated residuals between the items “number of teeth” and “loose teeth” (modification indices = 26.5). The resulting model with correlated residuals yielded an acceptable model fit (RMSEA = 0.06, CFI = 0.94, TLI = 0.90, WRMR = 0.77).

### Results from the binary models

Six variables (number of teeth, receding jaw bone, receding gums, presence of gum pockets, loose teeth, tooth extractions because of inflammation and deep gum pockets) were identified as having a prognostic value for PD. These variables and three additional demographic variables (age, sex and formal education) were used to calculate several binary scores for the assessment of PD. As there are three possible levels of disease severity, several results have to be considered for every possibility to classify patients with the score.

The item which correlated best with the different assessments of PD was the number of the remaining teeth. Therefore, the first proposed possibility to classify patients was to use only age and the self-reported number of the remaining teeth. This resulted in a sensitivity of 86/80/86% (no versus mild, moderate or severe PD/no or mild versus moderate or severe PD/no, mild or moderate versus severe PD), a specificity of 49/64/78% and an AUC of 0.73/0.78/0.86 (Table 3). For all models, the bias-corrected AUCs are only marginally lower. The AUCs of the models based on the dentist’s assessment applied to the radiograph scoring data differ most from the original model for the two models in which severe PD is detected (0.66 versus 0.85 and 0.77 versus 0.90).

**Table 3:** Diagnostic properties of logistic regression models

Model	Severity of detected PD	Reference standard: dentist’s assessment			Bias-corrected AUC	AUC of the original model applied on radiograph scoring data
		Sensitivity (%)	Specificity (%)	AUC		
Age + number of teeth	Mild, moderate or severe versus no	86.0	48.6	0.73	0.73	0.82
	Moderate or severe versus no or mild	80.3	64.1	0.78	0.77	0.72
	Severe versus no, mild or moderate	86.1	78.1	0.86	0.85	0.66
Age + 6 patient-reported items	Mild, moderate or severe versus no	64.2	88.5	0.82	0.81	0.88
	Moderate or severe versus no or mild	72.8	80.7	0.85	0.83	0.83
	Severe versus no, mild or moderate	96.6	81.5	0.92	0.90	0.77

Sensitivities, specificities and AUCs to detect different levels of severity of PD in the simple model and in the model including six questionnaire items. The table also shows the AUCs of these models after correction for overoptimism with bootstrap methods and the AUCs of the models if the independent assessment of PD with radiographs is used as a reference standard

When using all items from the patient questionnaire that were identified as being useful for the classification of PD, all models improved the diagnostic properties compared to the simple model only using age and the number of teeth. The AUCs of these models range between 0.82 and 0.92 depending on the severity level of PD that shall be detected. The models including sex and formal education of the patients did not show more favourable properties than those only including age as a demographic variable (data not shown). Therefore, sex and formal education were not included in the score.

### Results from the ordinal regression model

For the ordinal regression model, there was again a simple version with only the number of teeth and age, and one model including the five additional patient-reported items mentioned above. A likelihood ratio test showed that the model with the additional items is better than the simple version.

The following were the results for the score:

PD score = 2.8 + 0.033 × age + 0.37 × gum pockets + 0.30 × receding gums + 0.45 × loose teeth + 0.84 × receding jaw bone − 0.40 × tooth extractions − 0.12 × number of teeth.

The cut-offs were 1.83 for mild PD, 3.91 for moderate PD and 6.26 for severe PD. For example, a patient with an age of 40 years, no reported tooth or gum problems and all 28 teeth would have a score of 2.8 + 1.32–3.36 = 0.76 and would be classified as having no PD.

The following were the results for the simple version of the score:

PD score (simple version) = 2.5 + 0.036 × age − 0.11 × number of teeth. The corresponding cut-off values were 1.16 for mild PD, 2.88 for moderate PD and 4.91 for severe PD.

Table 4 shows the classification of the patients by the score compared to the reference standard. Patients with severe PD are only detected in less than 30% of the cases and most often classified as “moderate” (Table 5).

**Table 4:** Concordance of score and dentist’s assessment

Classification of PD by score (age + 6 patient-reported items)	Dentist’s assessment of PD				
	No	Mild	Moderate	Severe	Total
No	55	29	2	0	86
Mild	32	51	34	2	119
Moderate	9	21	37	19	86
Severe	0	0	1	8	9
Total	96	101	74	29	300

Comparison of classification of PD with the help of the PD score and the reference standard

**Table 5:** Diagnostic properties for ordinal regression model

Model	Severity of detected PD	Sensitivity (%)	Specificity (%)
Age + number of teeth	Mild, moderate or severe versus no	91.7	39.6
	Moderate or severe versus no or mild	49.5	85.8
	Severe versus no, mild or moderate	20.7	100
Age + 6 patient-reported items	Mild, moderate or severe versus no	84.8	57.3
	Moderate or severe versus no or mild	63.1	84.8
	Severe versus no, mild or moderate	27.6	99.6

Sensitivities and specificities for the detection of different levels of PD with the score derived from the ordinal regression model

The longer version of the score had considerably more specificity for the detection of PD than the short version (57% versus 40%). It also had a higher sensitivity for detecting moderate or severe PD.

## Discussion

A patient-reported questionnaire to detect PD in patients with RA was developed. Six patient-reported items were selected to build the age-adjusted score. The score had a fair sensitivity to detect mild, moderate or severe versus no PD and was very specific at excluding severe PD. Additionally, a simple score including only age and the number of teeth was evaluated. This score might be useful if PD shall be studied in a setting where it is only feasible to ask one additional question considering PD. The simple version also had a high sensitivity for detecting at least mild PD and a very good specificity to exclude severe PD. The overall properties of the score with six patient-reported items were more favourable than those of the simple score. The AUCs of 0.81, 0.83 and 0.90 respectively for the detection of at least mild, at least moderate or severe PD were comparable to those found by Dietrich et al. [11], Gilbert and Litaker [10] and Taylor and Borgnakke [17] and a little bit higher than those found by Genco et al. [9] (AUC of 0.76 for the detection of severe PD in the myocardial infection periodontitis study). In contrast to these questionnaires for self-reported PD, our score does not include sex or formal education. This might be due to the different study collectives with this study only including RA patients and the other studies including patients from the general population those who had a myocardial infarction.

Compared to the questionnaire used by Coburn et al. [13] that was also evaluated on RA patients, our questionnaire had a better AUC for the detection of severe PD (0.79 versus 0.90). For the detection of mild or moderate PD, the AUCs were comparable. In the investigation by Coburn et al., patients received a full-mouth periodontal examination to determine their PD status, while in the CAPEA periodontitis project, the patients’ dentists were asked to grade the severity of their patient’s PD semiquantitatively. This shows that in a setting where the diagnosis for PD was more standardised and clinically evaluated, the resulting PD score still does not have more favourable properties.

The items included in this score had some overlap with those identified by Dietrich et al. [11] (loosening of teeth, dentist told patient had lost bone around his or her teeth) but also included the presence of gum pockets and bleeding gums which are not represented in the final models of Dietrich et al., Taylor and Borgnakke [17] and Gilbert and Litaker [10] (in Gilbert’s score, a more general rating of “gum health” is included, though). There was also an overlap with the items used by Coburn et al. [13]. Items concerning bleeding gums, bone loss, deep pockets, loose teeth and oral surgery were also included in our questionnaire in a similar way. While “bleeding gums” was not included in the final PD score in our analysis; the parameter correlating best with PD in our analysis (number of teeth) was not included in Coburn et al.’s questionnaire.

One limitation of this study is that our reference standard to determine a patient’s PD status is the report of the patient’s individual dentist and was not evaluated by a study dentist. To validate the diagnosis, the radiographs were assessed externally by three independent dentists. If the PD score we developed is applied to these data, the AUCs are in the range of 0.77 to 0.88 which means that if an objective blinded assessment of PD is used as a reference standard, the questionnaire also performs reasonably well. While there were more male than female patients participating in the Coburn study, CAPEA patients form a representative sample of early arthritis patients in Germany with more female patients.

The sensitivity and specificity of the CAPEA PD questionnaire are reasonably good. In order to conduct large epidemiologic trials that further investigate the relationship between RA and PD, instruments with a high accuracy would be needed. The misclassification rate might be too high to assess the relationship between clinical features of RA and periodontal status, if the periodontal status is determined through a patient-reported questionnaire alone. This problem could partly be solved by using a continuous measure of PD instead of categorising patients to “no”, “mild”, “moderate” or “severe” PD. Using the PD score as a continuous measure would still allow investigating the correlation between the severity of clinical measures of RA and PD with less misclassification errors than when using the categorisation.

## Conclusions

The CAPEA PD score can be used as a measure of PD in epidemiological settings. In a categorical analysis using cut-off values, researchers should keep in mind, however, that this score does show only moderate diagnostic properties. If high accuracy is not essential, the number of teeth and age alone can also be used as a simple measure for the detection of the frequency of PD in patients with RA.

## Data Availability

The datasets analysed during the current study are not publicly available, because we respect our patient’s right to privacy. Consent for publication of the dataset has not been asked at the point of recruitment to the trial.

## Abbreviations

Anti-CCP: Anti-citrullinated protein
AUC: Area under the receiver operator characteristic curve
CAPEA: Course And Prognosis of Early Arthritis
PD: Periodontitis
CFA: Confirmatory factor analysis
CFI/TLI: Comparative fit index/Tucker-Lewis index
CRP: C-reactive protein
DAS28: Disease Activity Score including 28 joints
ESR: Erythrocyte sedimentation rate
RF: Rheumatoid factor
RMSEA: Root mean square error of approximation
SJC: Swollen joint count
TJC: Tender joint count
WRMR: Weighted root mean square residual
